# Sesamin: A Naturally Occurring Lignan Inhibits CYP3A4 by Antagonizing the Pregnane X Receptor Activation

**DOI:** 10.1155/2012/242810

**Published:** 2012-05-07

**Authors:** Yun-Ping Lim, Chia-Yun Ma, Cheng-Ling Liu, Yu-Hsien Lin, Miao-Lin Hu, Jih-Jung Chen, Dong-Zong Hung, Wen-Tsong Hsieh, Jin-Ding Huang

**Affiliations:** ^1^Department of Pharmacy, College of Pharmacy, China Medical University, Taichung 40402, Taiwan; ^2^Department of Emergency, Toxicology Center, China Medical University Hospital, Taichung 40447, Taiwan; ^3^Department of Food Science and Biotechnology, National Chung Hsing University, Taichung 40227, Taiwan; ^4^Graduate Institute of Pharmaceutical Technology, Tajen University, Pingtung 90741, Taiwan; ^5^School of Medicine and Graduate Institute of Basic Medical Science, China Medical University, Taichung 40402, Taiwan; ^6^Institute of Biopharmaceutical Sciences, Medical College, National Cheng Kung University, Tainan 70101, Taiwan; ^7^Department of Pharmacology, Medical College, National Cheng Kung University, Tainan 70101, Taiwan

## Abstract

Inconsistent expression and regulation of drug-metabolizing enzymes (DMEs) are common causes of adverse drug effects in some drugs with a narrow therapeutic index (TI). An important cytochrome, cytochrome P450 3A4 (CYP3A4), is predominantly regulated by a nuclear receptor, pregnane X receptor (PXR). Sesamin, a major lignan constituent in sesame seeds and oil, exhibits a variety of biological functions; however, the effect of sesamin on the modulation of CYP3A4 is not well understood. In this study, the effects of sesamin on the PXR-CYP3A4 pathway were characterized, as well as the underlying mechanisms of those effects. Sesamin potently attenuated CYP3A4 induction in a dose-dependent manner by blocking the activation of PXR. The PXR inducer-mediated inhibition of CYP3A4 was further evidenced by the ability of sesamin to attenuate the effects of several PXR ligands in the CYP3A4 reporter assay. Further mechanistic studies showed that sesamin inhibited PXR by interrupting the interacting with coregulators. These results may lead to the development of new therapeutic and dietary approaches to reduce the frequency of inducer-drug interaction. Sesamin was established as a novel inhibitor of PXR and may be useful for modulating DMEs expression and drug efficacies. Modification of CYP3A4 expression and activity by consumption of sesamin may have important implications for drug safety.

## 1. Introduction

Sesame seeds (*Sesamum indicum*) and their oil have been used in human diets for thousands of years and are believed to provide health benefits. A major constituent of sesame is a lignan called sesamin. Sesamin has a variety of biological functions including reduction of serum and hepatic cholesterol levels [1–5] as well as serum triglycerides [6] by increasing hepatic fatty acid oxidation [1, 2]. Sesamin's involvement in the suppression of hypertension [7, 8] and stroke prevention has been extensively studied by many researchers [9]. Moreover, sesamin has been shown to elevate the levels of *γ*-tocopherol [10]; decrease production of endotoxin-induced interleukin (IL)-1*β*, prostaglandin E2 (PGE2), and thromboxane B2 [11]; elevate the production of IL-6 [12], thus inhibiting endotoxin-mediated shock [11]. Some studies also suggest that sesamin has an inhibitory effect on 7,12-dimethylbenz[a]anthracene-induced mammary carcinogenesis [13] and inhibits the growth of a variety of neoplastic cells through different mechanisms [14–18]. Thus, sesamin is believed to protect against cancer and other chronic diseases.

Cytochrome P450- (CYP450-) dependent monooxygenase occupies a pivotal role in the metabolism and detoxification process of endogenous and exogenous compounds [19]. CYP3A4 is the most abundant CYP450 and has clinical importance because it metabolizes numerous pharmaceutical agents [19]. It is highly expressed in the liver and intestine and represents 40% of the total hepatic and 80% of the total intestinal CYPs [20, 21]. CYP3A4 is also involved in the metabolism of endogenous substrates (retinoic and bile acids) and steroidal hormones (testosterone and estrogen) [22]. In addition, dietary and environmental chemicals such as aflatoxin B1 and some herbicides are also CYP3A4 substrates [23, 24]. The expression and activity of CYP3A4 are greatly impacted by many drugs and dietary chemicals such as rifampin (an antibiotic), carbamazepine (an anticonvulsant), glucocorticoids, and hyperforin (a major component of St. John' wort) [25]. Individual variations in drug metabolism may be due to the differential expression of the CYP3A4 enzyme, induced by some of these compounds [20]. CYP3A4 protein expression in hepatocytes varies more than 50 folds among individuals, and the enzymatic activity varies by at least 20 folds [26]. Changes caused by drug-metabolizing enzymes (DMEs) are common and undesirable, and these enzymes influence the therapeutic effects of drugs, particularly those having a narrow therapeutic index [27]. Since CYP3A4 activity may be affected by some xenobiotics, it plays a crucial role in drug-drug interactions. For example, coadministration with rifampin, phenytoin, or carbamazepine may reduce plasma concentrations of a broad range of CYP3A4 drug substrates to less than a half [28].

Compounds that modulate the upper control element of CYP3A4, human pregnane X receptor (PXR), can also modulate CYP3A4 expression and drug metabolism activity [29–31]. Human PXR is a member of the nuclear receptor (NR) superfamily, encoded by the *NR1I2* gene [29–33]. PXR regulates the expression of many enzymes involved in the metabolism of xenobiotic and endobiotic compounds such as CYP2B, CYP2C, CYP3A, glutathione *S*-transferases (GSTs), sulfotransferases (SULTs), and uridine diphosphate (UDP)-glucuronosyltransferases [34–37]. Beside phase I and phase II drug metabolizing enzymes, PXR also regulates the expression of phase III enzymes, including the drug transporters OATP2, MDR1, MRP2, and MRP3 [38, 39]. Being a ligand-activated transcription factor, PXR is activated by a vast array of natural and synthetic compounds, including the antibiotic rifampin [31], the antihypertension drug nifedipine, the antimycotic drug clotrimazole [31, 32], the antiglucocorticoid RU-486 [32], and a variety of naturally occurring steroids.

The PXR pathway is also activated by a large number of prescription drugs designed to treat infection, cancer, convulsions, and hypertension and is believed to play an important role in drug metabolism and efflux as well as inducer-drug interactions [40]. PXR increases the transcription of CYP3A4 as well as other DMEs and transporters after exposure to its agonists [41, 42]. Because many anticancer agents have narrow therapeutic indices, modulation of the PXR-CYP3A4 pathway may cause unpredictable efficacy.

Because the PXR-CYP3A4 pathway is extremely important in drug efficacy, it is very important to find an agent that may reduce the effect of inducer-drug interaction. PXR antagonists may be useful for the study of the molecular basis of receptor function. Although many PXR agonists have been reported, comparatively few PXR compounds antagonizing the PXR-CYP3A4 pathway have been identified [43–47].

In this study, the effects of sesamin on the PXR-CYP3A4 pathway and the underlying mechanisms were characterized. Sesamin potently attenuated CYP3A4 induction in a dose-dependent manner by blocking the activation of nuclear receptors, especially PXR through CYP3A4 reporter assay. The PXR inducer-mediated inhibition of CYP3A4 was further evidenced by the ability of sesamin to attenuate the effects of rifampin, paclitaxel, and SR12813 in the CYP3A4 reporter assay. Further mechanistic studies showed that sesamin inhibited PXR by interrupting the binding of steroid receptor coactivator-1 (SRC-1) and hepatocyte nuclear factor 4*α* (HNF4*α*). Our results may lead to the development of important new therapeutic and dietary approaches to reduce the frequency of undesirable drug interactions. Here, we established sesamin as a novel and natural potent inhibitor of PXR and proved that it can be a useful tool for modulating DMEs expression. Modification of CYP3A4 by consumption of sesamin could have important implications for drug safety.

## 2. Materials and Methods

### 2.1. Chemicals and Cell Cultures

 Rifampin (RIF), sesamin (SSM), 5-pregnen-3*β*-ol-20-one-16*α*-carbonitrile (PCN), 6-(4-chlorophenyl)imidazo [2,1-b][1,3]thiazole-5-carbaldehyde O-(3,4-dichlorobenzyl)oxime (CITCO), SR12813, and paclitaxel were purchased from Sigma-Aldrich (St. Louis, MO, USA) and dissolved in DMSO at concentrations appropriate for the specific studies in which they were used. HepG2 and LS174T cells were purchased from the Food Industry Research and Development Institute (FIRDI, Taiwan, ROC) and maintained in a minimum essential medium (MEM) *α* medium supplemented with 10% fetal bovine serum without antibiotics, in a 5% CO_2_ atmosphere at 37°C.

### 2.2. Plasmids Construction

 Plasmids pcDNA3-PXR and pGL3B-CYP3A4 [(−444/+53)(−7836/−7208)], containing full-length human PXR and CYP3A4 promoter constructs, respectively, have been described previously [48]. Full-length SRC-1 plasmids were kindly provided by Lih-Yuh Chen Wing (Department of Physiology, National Cheng Kung University, Tainan, Taiwan). A fragment encoding residues 595–800 of the human SRC-1 (GenBank accession number U90661) receptor interacting domain (RID) and the full-length PXR were cloned into the pBIND-GAL4 and pACT-VP16 vectors to prepare pBIND-SRC-1 and pACT-PXR, respectively, as described previously [49]. The expression plasmids pcDNA3-HNF4*α*, pBIND-PXR, and pACT-HNF4*α* were prepared as described previously [50]. A full-length human constitutive androstane receptor (CAR) cDNA (GenBank accession number NM_001077480) was purchased from Open Biosystems (Huntsville, AL, USA), and full-length rat PXR (GenBank accession number NM_052980) was cloned from rat liver cDNA. Both gene products were amplified from cDNA (for human CAR: forward primer, 5′-AAG GAT CCA CGT CAT GGC CAG TAG-3′; reverse primer, 5′-CCA ATC TAG AGC ATT TTC CCA CTC-3′; for rat PXR: forward primer, 5′-GAT GGG ATC CTG GAG ATG AGA CCT GAG G-3′; reverse primer, 5′-CTC ATC TAG AGC CAC TCA GCC GTC CGT G-3′). The polymerase chain reaction (PCR) product was digested with *Bam*HI and *Xba*I, and restriction enzyme cut sites were introduced into the primers before performing PCR. The cut fragments were cloned into the pcDNA3 vectors with corresponding restriction enzyme sites to generate pcDNA3-CAR and pcDNA3-rPXR. The reporter construct pG5luc and an internal control plasmid, pRC-CMV-*β*-galactosidase, were purchased directly from Promega (Madison, WI, USA) and Invitrogen (Groningen, Netherlands), respectively.

### 2.3. Assessment of Cell Cytotoxicity

To verify ≥80% cell viability following xenobiotic exposure, cell viability was evaluated using the modified acid-phosphatase (ACP) assay, with *p*-nitrophenyl phosphate (PNPP) disodium salt as a substrate. The cell culture media were aspirated, and the cells were washed with phosphate-buffered saline (PBS). Following the wash, 100 *μ*L of the ACP reagent [(0.1 M sodium acetate (pH 5.5), 0.1% Triton X-100, and 10 mM PNPP)] were added. After 1 h of incubation at 37°C, the enzyme activity was stopped by adding 10 *μ*L of 1 N NaOH, and the extent of activity was determined photometrically at a wavelength of 405 nm [50].

### 2.4. Determination of CYP3A4 Enzymatic Activity

CYP3A4 enzyme activity was measured using the P450-Glo assay (Promega). This assay measures CYP3A4 enzyme activity by using luciferin-PFBE as a specific substrate for CYP3A4. Briefly, cells (1 × 10^5^/well) were seeded into a 48-well plate and treated with various concentrations of sesamin alone or in combination with 20 *μ*M rifampin. After 48 h of incubation, the medium was discarded, and the cells were rinsed twice with PBS. Fresh complete medium, containing 50 *μ*M luciferin-PFBE, was added to the cells and incubated for 4 h at 37°C. Subsequently, 50 *μ*L of the medium from each well was transferred to a white, opaque, 96-well plate, and 50 *μ*L of the reconstituted luciferin detection reagent was added to initiate a luminescent reaction. After a 20-min stabilization period, luminescence was read through a multi-mode microplate reader (Synergy HT, BioTek Instruments, Inc.; Winooski, VT, USA). Fold changes of post-treatment luminescence were calculated by dividing the detected luminescence signals by values reflecting cell viability.

### 2.5. Real-Time Polymerase Chain Reactions (Real-Time PCR)

The total RNA in cell cultures was extracted with Rezol C&T RNA extraction reagent (Protech Technologies, Inc., Taiwan), and 1 *μ*g of total RNA was reverse transcribed using oligo-dT (Promega) as a primer in 20 *μ*L of reverse transcription solution, containing 1 *μ*L of reverse transcriptase (Promega). Real-time PCR was performed using a Corbett instrument (QIAGEN; Hilden, Germany) and SYBR Green Master Mix (Protech Technologies, Inc., Taiwan), according to the manufacturer's instructions. In all real-time PCR experiments, both a nontemplate control (NTC) and a standard curve were amplified. The RNA abundance was normalized to *β*-actin (a house-keeping gene) RNA in each sample. The primers and PCR conditions used in this study are shown in Table 1.

### 2.6. Western Blotting

Protein expressions of CYP3A4, PXR, 9-*cis* retinoic acid receptor (RXR*α*), and HNF4*α* were measured by using western blotting. HepG2 cells were seeded at a density of 2 × 10^6^ cells/10-cm dish, before drug treatment. Various concentrations of sesamin, alone or in combination with 20 *μ*M rifampin, were added to the HepG2 cell culture for 48 h. Following drug treatment, the medium was removed, and the cells were rinsed twice with ice-cold PBS. After adding 200 *μ*L of ice-cold RIPA buffer with protease inhibitors, the cells were scraped from the surface of the culture dish and allowed to stand on ice for 20 min, and the cell lysates were centrifuged (14,000 rpm for 30 min at 4°C). Total protein (50 *μ*g) from the supernatant was resolved by using 10% sodium dodecyl sulfate-polyacrylamide gel electrophoresis (SDS-PAGE) and transferred onto nitrocellulose (NC) paper. These blots were exposed to primary antibodies against CYP3A4, PXR, RXR*α*, HNF4*α*, and *β*-actin (Santa Cruz Biotechnology Inc., Santa Cruz, CA, USA). The relative density of the immunoreactive bands was quantitated using densitometry (ImageQuant LAS 4000, GE Healthcare, Waukesha, WI, USA) following detection using an enhanced chemiluminescence detection system (Millipore, Billerica, MA, USA).

### 2.7. Transfection, CYP3A4 Reporter Assay, and Mammalian Two-Hybrid Assay

 HepG2 and LS174T cells were plated in 96-well plates (Nunc, Rochester, NY, USA), at a density of 1.8 × 10^4^ cells/well, 18 h before transfection. Plasmid DNA was introduced using PolyJET transfection reagent (SignaGen Laboratories, Rockville, MD), according to the manufacturer's instructions. For the CYP3A4 reporter assay, 0.15 *μ*g of a CYP3A4 reporter construct, 0.02 *μ*g of control *β*-galactosidase plasmid, and either 5 ng PXR/CAR/rPXR or 0.04 *μ*g SRC-1/HNF4*α* expression plasmid was added per well. For the mammalian two-hybrid assays, transfection was carried out by mixing 0.10 *μ*g of the pG5luc reporter gene, 0.04 *μ*g each of the pBIND-GAL4 and pACT-VP16 constructs, and 0.02 *μ*g of the control *β*-galactosidase plasmid per well. After 6 or 7 h, the cells were exposed to rifampin, sesamin, CITCO, PCN, SR12813, paclitaxel, or a similar volume of DMSO. After a further 20–24 h of incubation, the cells were lysed *in situ* with 80 *μ*L of cell culture lysis reagent (Promega). The cleared lysate (30 *μ*L) was used for the *β*-galactosidase assay. A 50-*μ*L aliquot of each cleared lysate was used for the reporter assay, following which 80 *μ*L of luciferase assay reagent (Promega) was added to the lysates. Luminescent signals were measured using a luminescence multi-mode microplate reader. Luciferase activities were normalized to the corresponding *β*-galactosidase activity. Data shown are means of triplicate or quadruplicate transfections ± standard deviations, as indicated.

### 2.8. Statistical Analysis

All experiments were repeated at least thrice independently. Values, expressed as mean ± SD, were analyzed using one-way ANOVA followed by Tukey's test for multiple comparisons. All statistical analyses were performed using SPSS for Windows, version 17.0 (SPSS, Inc., Chicago, IL, USA). A *P* value < 0.05 was considered statistically significant.

## 3. Results

### 3.1. Cell Viability of HepG2 and LS174T Cells following Exposure to Sesamin

**Figure 1:**
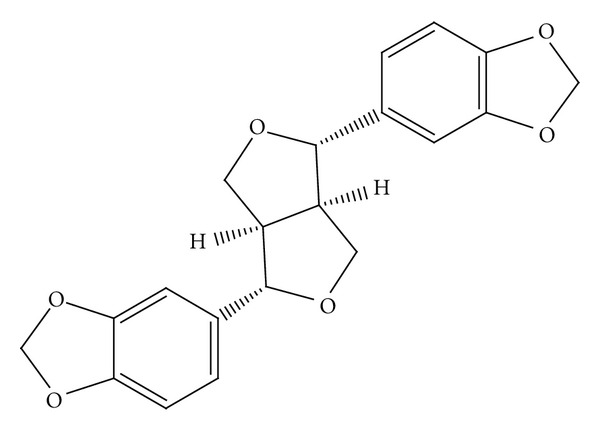
The chemical structure of sesamin.

**Figure 2:**
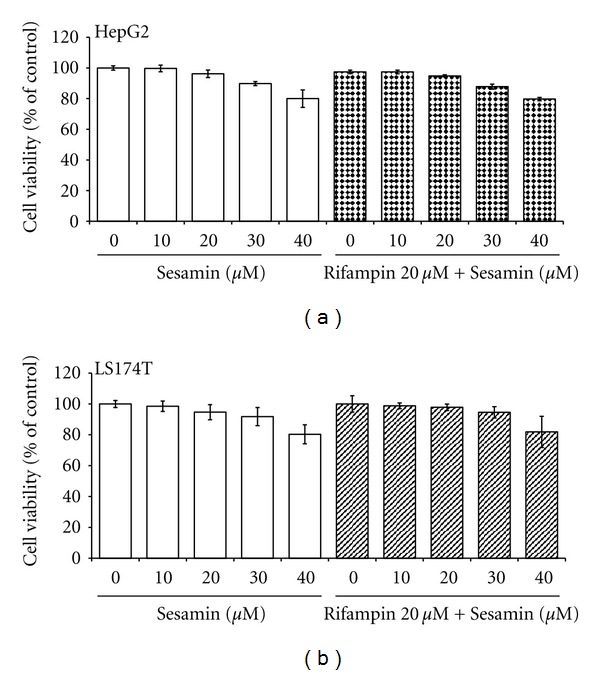
Cell viability of HepG2 and LS174T cells following exposure to sesamin alone and in combination with rifampin. (a) HepG2 and (b) LS174T cells were exposed to sesamin (10–40 *μ*M), rifampin (20 *μ*M), and combinations of rifampin (20 *μ*M) and sesamin (10–40 *μ*M) for 48 h. Cell viability was monitored by cellular acid phosphatase activity using PNPP as a substrate. Results are presented as mean ± SD (*n* = 4).

 Since sesamin (Figure 1) has been shown to inhibit proliferation of multiple types of malignant cells [13–17], a cell viability test was performed to rule out cytotoxic effects due to sesamin. As shown in Figure 2, HepG2 (Figure 2(a)) and LS174T (Figure 2(b)) cells were exposed to a range of concentrations of sesamin alone and in combination with rifampin for 48 h, and the cell viability was assessed using the ACP assay. Rifampin did not show cytotoxicity toward either cell line. Sesamin caused mild cytoxicity as compared to DMSO-treated cells. However, even after exposure to 40 *μ*M sesamin (the highest concentration tested), cell viability remained approximately 80%.

### 3.2. Sesamin Inhibits Rifampin-Induced CYP3A4 Enzyme Activity, mRNA, and Protein Expression in HepG2 Cells

**Figure 3:**
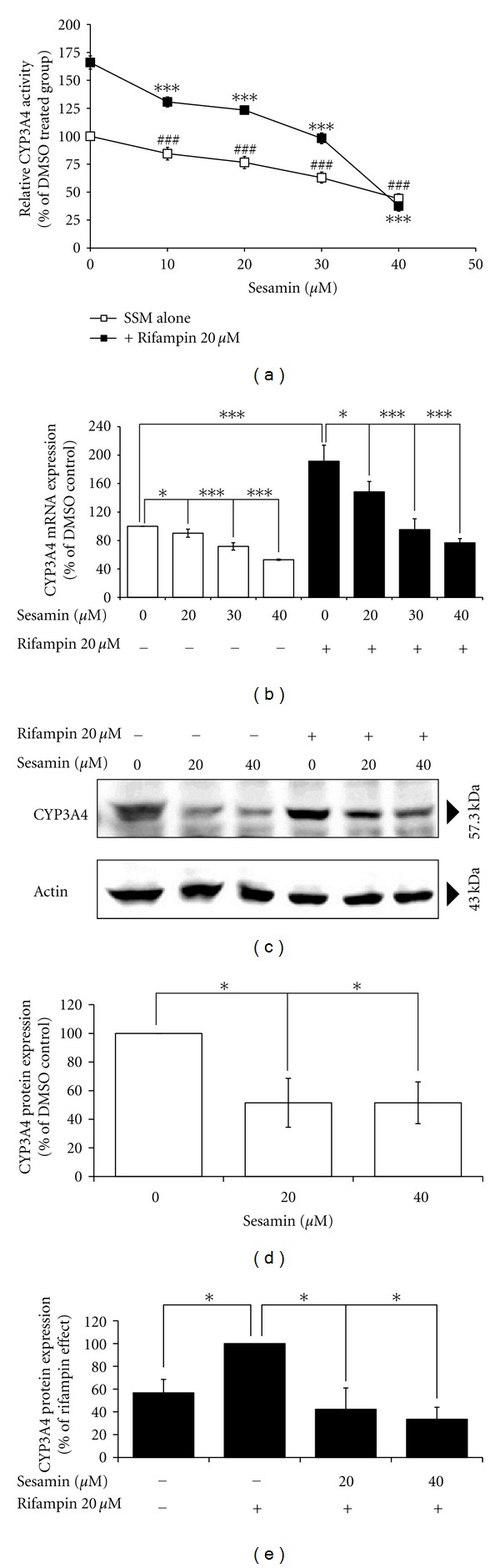
Effect of sesamin (SSM), alone or sesamin in combination with rifampin, on CYP3A4 enzyme activity, mRNA expression, and protein expression. (a) Various concentrations of sesamin, alone or in combination with 20 *μ*M rifampin, were added to cultured HepG2 cells for 48 h. CYP3A4 enzyme activities were measured by using P450-Glo assays with 50 *μ*M luciferin-PFBE as a substrate. Values are presented as mean ± SD (*n* = 3); ^###^ *P* < 0.001 and ***P < 0.001 as compared to DMSO-treated or 20-*μ*M rifampin-treated cells, as appropriate. (b) HepG2 cells were treated with sesamin and rifampin, individually or in combination, for 48 h, mRNA was collected, and the expression of CYP3A4 and an internal control, *β*-actin, were analyzed using RT-PCR. Values were normalized relative to the expression of *β*-actin, with the CYP3A4 expression of DMSO-treated cells set at 100%. The results are as expressed as mean ± SD (*n* = 3) of the relative expression of CYP3A4; *P < 0.05 and ***P < 0.001 as compared to DMSO-treated or 20-*μ*M rifampin-treated cells, as indicated in the figure. (c) HepG2 cells were treated with sesamin and rifampin, individually or in combination, for 48 h. Whole cell extracts were harvested, and the expression of CYP3A4 and the internal control *β*-actin were analyzed using western blotting. (d) Quantitation of band intensity of the CYP3A4 protein was normalized to *β*-actin expression. The basal expression of the CYP3A4 protein was set at 100%. The values are presented as mean ± SD (*n* = 3); *P < 0.05 as compared to DMSO-treated cells. (e) Quantitation of band intensity of CYP3A4 protein was normalized to the *β*-actin expression. The rifampin-induced CYP3A4 protein expression was set at 100%, and the values are presented as mean ± SD (*n* = 3); *P < 0.05 as compared to rifampin-treated cells.

Sesamin's ability to inhibit the basal and rifampin-induced CYP3A4 enzyme activity as well as mRNA and protein expression was assessed by exposing HepG2 cells to concentrations of sesamin (10–40 *μ*M) alone or in combination with 20 *μ*M rifampin, for 48 h. As shown in Figure 3(a), treatment with 20 *μ*M rifampin increased CYP3A4 activity by 66%, as compared to the DMSO-treated controls. Treatment of HepG2 cells with 10, 20, 30, and 40 *μ*M sesamin for 48 h significantly decreased basal CYP3A4 enzyme activity by 15.6%, 23.4%, 37.1%, and 55.8% (*P* < 0.001), respectively, as compared to controls. Coincubation of cells with 20 *μ*M rifampin and the above-mentioned concentrations of sesamin significantly attenuated the rifampin-induced CYP3A4 enzyme activity. The inhibitory effect of sesamin was concentration dependent, decreasing CYP3A4 activity by 21.3%, 25.7%, 41%, and 77.3%, respectively, (*P* < 0.001), as compared to the rifampin-treated cells (Figure 3(a)).

 To determine if decreased CYP3A4 enzyme activity was a consequence of decreased mRNA expression, real-time PCR was used to assess CYP3A4 mRNA levels. Sesamin (20, 30, 40 *μ*M) was found to significantly decrease the basal CYP3A4 mRNA expression in HepG2 cells after incubation for 48 h by 9.67% (*P* < 0.05), 28.3% (*P* < 0.001), and 47% (*P* < 0.001), respectively, as compared to untreated cells (Figure 3(b)). Conversely, rifampin significantly induced CYP3A4 mRNA expression by 91.3% (*P* < 0.001). However, the rifampin-induced increase in CYP3A4 mRNA expression was decreased when sesamin (20, 30, and 40 *μ*M) was coadministered [26.1% (*P* < 0.05), 50.1% (*P* < 0.001), and 59.9% (*P* < 0.001), resp.]. Therefore, sesamin had significant effects on the basal and induced levels of CYP3A4 mRNA expression (Figure 3(b)).

To evaluate CYP3A4 protein expression, HepG2 cells were treated with 20 and 40 *μ*M sesamin alone or in combination with 20 *μ*M rifampin for 48 h. Protein expression levels were determined using western blotting. We found that sesamin (20 *μ*M) significantly decreased the basal level of CYP3A4 protein expression (48.5%, *P* < 0.05) as compared to the DMSO-treated group (Figures 3(c) and 3(d)). Rifampin significantly enhanced CYP3A4 protein expression by 55.5% (*P* < 0.05). Coincubation of the cells with 20 and 40 *μ*M sesamin with 20 *μ*M rifampin significantly decreased rifampin-induced CYP3A4 protein expression by 57.7% (*P* < 0.05) and 66.5% (*P* < 0.05), respectively, as compared to rifampin-induced cells. The effects were concentration dependent (Figures 3(c) and 3(e)) and consistent with those from the mRNA expression assays. Thus, sesamin inhibits CYP3A4 enzyme levels by reducing expression of both mRNA and protein.

### 3.3. Sesamin Inhibits PXR-Mediated CYP3A4 Promoter Activity in HepG2 and LS174T Cells

**Figure 4:**
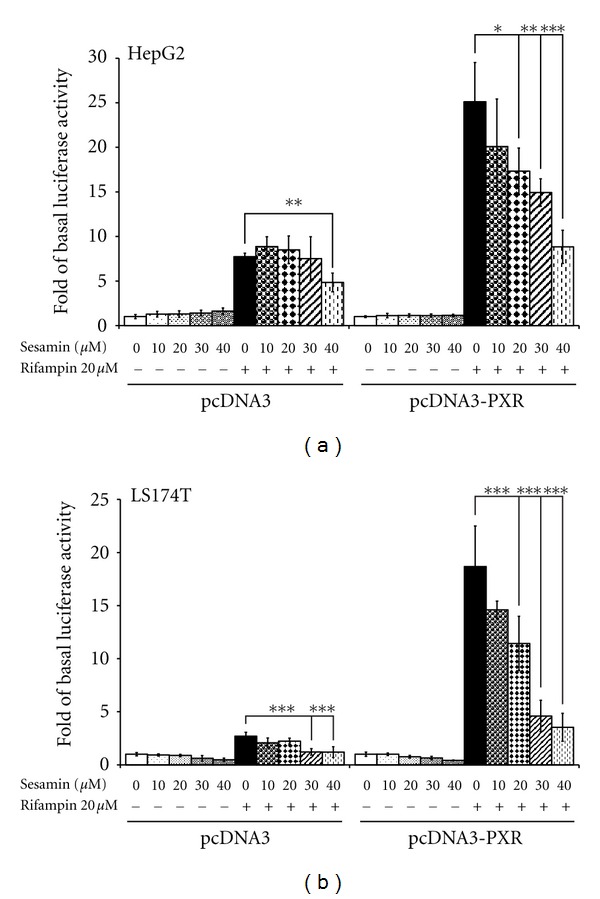
Transient transcription assays of CYP3A4 reporter activity in HepG2 and LS174T cells to determine the effects of sesamin- or rifampin-mediated activation. (a) HepG2 and (b) LS174T cells were cotransfected with a vector control (pcDNA3) or PXR expression plasmid (pcDNA3-PXR), a CYP3A4 luciferase reporter plasmid, and a pRC-CMV-*β*-galactosidase vector. Subsequently, the transfected cells were exposed to sesamin and/or rifampin for 24 h. The results are expressed as mean ± SD (*n* = 4); *P < 0.05; **P < 0.01; ***P < 0.001, as compared to rifampin-treated cells.

PXR is a main ligand-activated transcription factor of CYP3A4. PXR transactivation reporter assays were conducted to assess the inhibitory effect of sesamin alone or with rifampin-induced PXR transactivation activity, on the CYP3A4 promoter. Cells were cotransfected with a *β*-galactosidase expression vector, a reporter gene construct containing a responsive element for PXR in the CYP3A4 promoter, and a human PXR expression plasmid or vector control. Cells were treated with different concentrations of sesamin, with or without 20 *μ*M rifampin. The luciferase activity was measured after 24 h of drug treatment. The results are shown in Figure 4. In HepG2 cells, sesamin (20, 30, and 40 *μ*M) strongly attenuated the transactivation of the CYP3A4 promoter induced by rifampin in a concentration-dependent manner when cotransfected with human PXR. As shown in Figure 4(a), transactivation decreased by 31% (*P* < 0.05), 40.6% (*P* < 0.01), and 64.8% (*P* < 0.001), respectively, as compared to the rifampin-induced groups. Similar experiments were also performed using a colon adenocarcinoma cell line (LS174T) to exclude the possibility of a cell-type-specific effect. As shown in Figure 4(b), similar results were found when these cells were transfected with PXR in the presence of rifampin and sesamin. Treatment of LS174T cells with sesamin for 24 h significantly attenuated PXR-mediated activation of the CYP3A4 promoter induced by rifampin; the effect of sesamin was concentration dependent, with 40 *μ*M sesamin producing the greatest inhibitory effect [80% decrease, (*P* < 0.001), as compared to rifampin treatment alone].

Endogenous PXR and CAR have been identified in HepG2 and LS174T cells [51, 52]; thus, rifampin could activate the CYP3A4 reporter response in cells carrying the control vector only. As shown in Figures 4(a) and 4(b), exposure of vector-transfected groups to 40 *μ*M sesamin significantly decreased rifampin-induced CYP3A4 promoter activity in HepG2 cells (a decrease of 37%, *P* < 0.01; Figure 4(a)) and in LS174T cells (a decrease of 55.7%, *P* < 0.001; Figure 4(b)), as compared to the rifampin-treated controls. In contrast, cells without rifampin induction did not show a significant reduction of CYP3A4 reporter activity in the presence of sesamin. These results suggest a PXR-dependent mechanism of regulation.

### 3.4. Inhibition of Human CAR- and Rat PXR-Mediated CYP3A4 Induction

**Figure 5:**
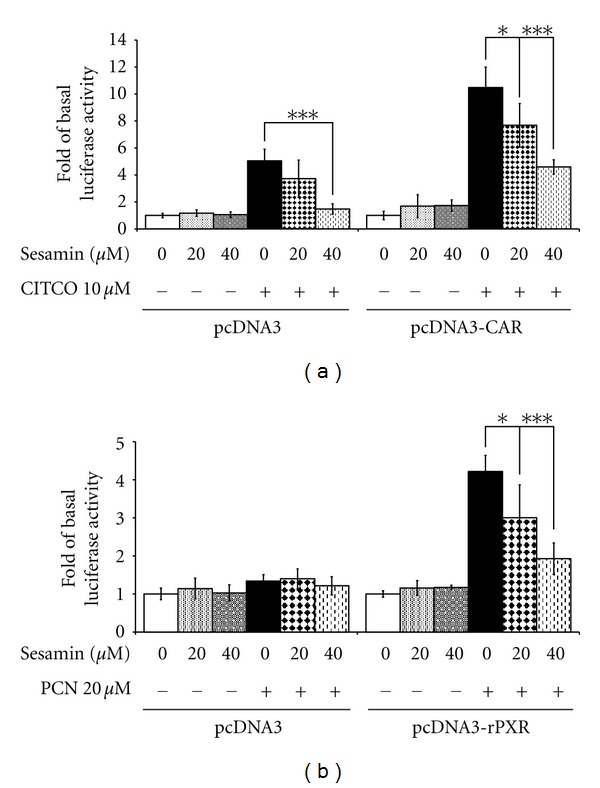
Transactivation of the CYP3A4 promoter by human CAR and rat PXR in the presence of sesamin and/or rifampin. HepG2 cells were cotransfected with a vector control (pcDNA3) and (a) human CAR expression plasmids (pcDNA3-CAR) or (b) rat PXR expression plasmids (pcDNA3-rPXR), a CYP3A4 luciferase reporter plasmid, and pRC-CMV-*β*-galactosidase vectors. Subsequently, the transfected cells were exposed to sesamin and/or rifampin for 24 h. The results are expressed as mean ± SD (*n* = 4); *P < 0.05 and ***P < 0.001, as compared to rifampin-treated cells.

CYP3A4 expression is also regulated by CAR at the same response elements in the CYP3A4 promoter [30]. Similar reporter assays were performed in HepG2 cells to evaluate the effect of sesamin on human CAR-mediated CYP3A4 transactivation. Sesamin (20 and 40 *μ*M) was found to markedly attenuate CITCO-induced CYP3A4 promoter activation through CAR (26.6%, *P* < 0.05, and 56.2%, *P* < 0.001, as compared to CITCO treatment alone, at 20 and 40 *μ*M sesamin, resp.; Figure 5(a)).

CYP3A4 inhibition is predominantly modulated by the effect of PXR on CYP3A4 expression and partly modulated by CAR. Previous reports have shown that the differences in the ligand-binding domain of human and rat PXR may lead to ligand-induced species-specific effects [53]. Therefore, sesamin inhibition of rat PXR activation was also tested. As shown in Figure 5(b), when rat PXR-transfected cells were treated with the rodent-specific ligand, PCN, CYP3A4 activity was enhanced by 322%, as compared to DMSO-treated controls. Sesamin strongly attenuated rat PXR-mediated CYP3A4 promoter activity with PCN induction by either 20 *μ*M sesamin (decreased by 28.9%, *P* < 0.05) or 40 *μ*M sesamin (decreased by 54.3%, *P* < 0.001), as compared to the PCN-treated cells. The results indicate that sesamin activity is not highly species specific.

### 3.5. Effects of Sesamin on PXR, RXR*α*, and HNF4*α* Protein Expression

**Figure 6:**
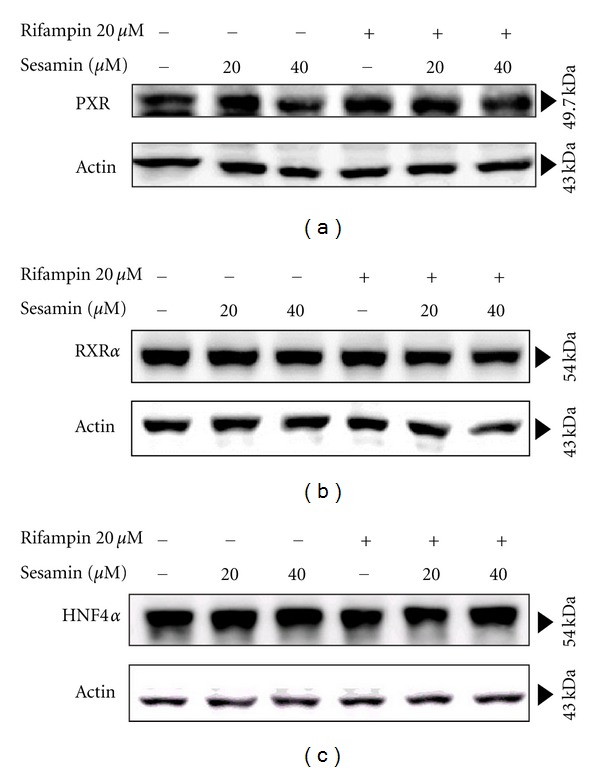
Effect of sesamin on basal and induced PXR, RXR*α*, and HNF4*α* protein expression. HepG2 cells were seeded in tissue-culture wells prior to being exposed to various concentrations of sesamin alone or in combination with 20 *μ*M rifampin for 48 h. Whole cell lysates were collected, and protein expression of (a) PXR, (b) RXR*α*, and (c) HNF4*α* were detected using western blotting.

 Based on the finding that sesamin reduces PXR activity in cell-based assays,* in vitro*, the impact of sesamin on PXR protein expression was investigated. PXR binds to DNA as a heterodimer with RXR*α*. The heterodimer can bind to and induce transcription from response elements located in the CYP450 genes [29–33]. In addition, rifampin-induced CYP3A4 requires PXR cross-talk with HNF4*α* on the CYP3A4 promoter transcription complex [54]. Western blots were performed to evaluate the PXR, RXR*α*, and HNF4*α* protein levels in sesamin-treated cells. HepG2 cells were treated with 20 and 40 *μ*M sesamin alone or in combination with 20 *μ*M rifampin and lysed after 48 h of exposure. As shown in Figure 6, none of the treatment conditions resulted in an alteration of protein expression of PXR (Figure 6(a)), RXR*α* (Figure 6(b)), or HNF4*α* (Figure 6(c)). The results indicated that the reduction of CYP3A4 activity was not due to decreased expression of the upper control elements.

### 3.6. Inhibition of Coregulation of Human SRC-1 and HNF4*α* with PXR by Sesamin

**Figure 7:**
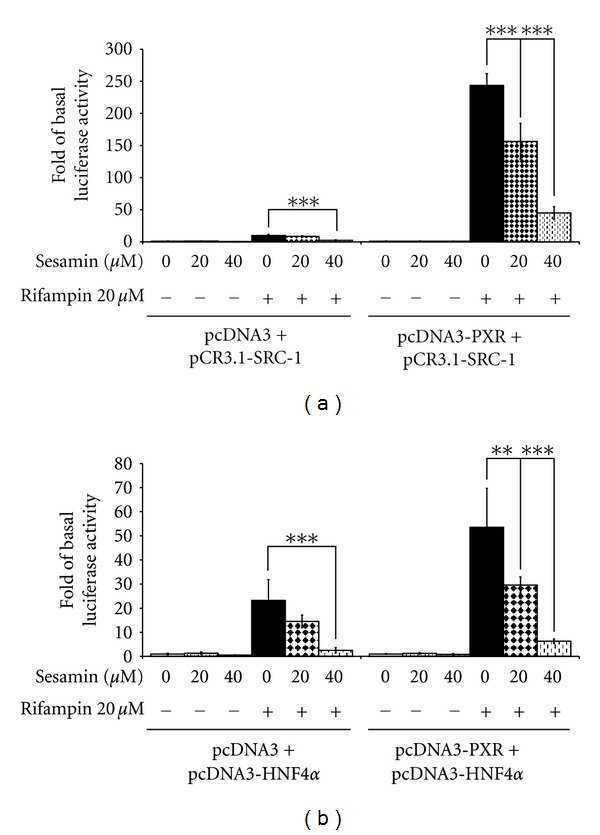
Coregulation of SRC-1 and HNF4*α* with PXR was disrupted by sesamin. HepG2 cells were cotransfected with (a) a vector control (pcDNA3) or PXR expression plasmid (pcDNA3-PXR) and SRC-1 expression plasmid (pCR3.1-SRC-1), or (b) a vector control (pcDNA3) or PXR expression plasmid (pcDNA3-PXR) and HNF4*α* expression plasmid (pcDNA3-HNF4*α*), in combination with the CYP3A4 luciferase reporter plasmid and the pRC-CMV-*β*-galactosidase vector. Subsequently, the transfected cells were exposed to sesamin and/or rifampin for 24 h. Luciferase activity was measured and normalized to the corresponding *β*-galactosidase activity. The results are expressed as mean ± SD (*n* = 4); ***P < 0.001, as compared to rifampin-treated cells.

 Earlier studies showed that cotransfection of SRC-1 and HNF4*α* into cells produced a synergistic increase in CYP3A4 promoter activity [49, 54, 55]. This part of the study sought to determine whether the effects of these two CYP3A4 promoter coregulators were altered by the presence of sesamin. Full-length human SRC-1 or HNF4*α* expression plasmids were cotransfected with full-length human PXR/vector control and CYP3A4 reporter constructs into HepG2 cells. The cells were then exposed to sesamin, alone or in combination with rifampin, followed by measurement of luciferase activity after 24 h of incubation. Since HepG2 cells contain endogenous PXR, cells transfected with the vector control and SRC-1 demonstrated a 98% increase in CYP3A4 promoter activity in the presence of rifampin (Figure 7(a)). Sesamin (40 *μ*M) decreased the promoter activity to 24.9% (*P* < 0.001) of the level observed in rifampin-treated cells. Transfection with PXR and SRC-1 expression plasmids resulted in a CYP3A4 promoter activity enhancement of 243.6 folds, in the presence of rifampin. Again, sesamin (20 and 40 *μ*M) strongly disrupted the enhancement of the promoter activity; activity was decreased to 64.2% (*P* < 0.001) or 18.5% (*P* < 0.001), respectively, of the level observed in rifampin-treated cells.

The coregulation of HNF4*α* and PXR was also evaluated. When cells were cotransfected with the vector control and full-length HNF4*α* prior to rifampin exposure, CYP3A4 activity was enhanced 23.2 folds. This effect was disrupted by sesamin, with the greatest inhibition at a sesamin concentration of 40 *μ*M (decreased by 89.3%, *P* < 0.001, as compared to rifampin-treated groups). The coregulation strength was not as great as that observed in the SRC-1/PXR cells; the presence of HNF4*α* and PXR enhanced CYP3A4 promoter activity by 53.6 folds. As Figure 7(b) shows, 20 and 40 *μ*M sesamin significantly reduced CYP3A4 promoter activity in these cells when exposed to rifampin (decreases of 44.8%, *P* < 0.01, and 88.3%, *P* < 0.001, resp., as compared to rifampin-treated groups). These results indicated that sesamin disrupts the coregulation effects of SRC-1/PXR and HNF4*α*/PXR on CYP3A4 promoter activity.

### 3.7. Sesamin Disrupts the Interaction of SRC-1/PXR and HNF4*α*/PXR in LS174T Cells

Ketoconazole has previously been shown to inhibit the expression of CYP3A4 by disrupting the interaction of SRC-1 and PXR as well as that of HNF4*α* and PXR [50]. To determine whether sesamin could inhibit SRC-1/PXR or HNF4*α*/PXR interaction, a GAL4/VP16 mammalian two-hybrid reporter system was used. The RID of SRC-1 and the full-length PXR gene were fused with the GAL4 DNA-binding domain to become pBIND-SRC-1 and pBIND-PXR, respectively. Similarly, the full-length PXR and HNF4*α* were fused with the VP-16 vector to become pACT-PXR and pACT-HNF4*α*, respectively.

**Figure 8:**
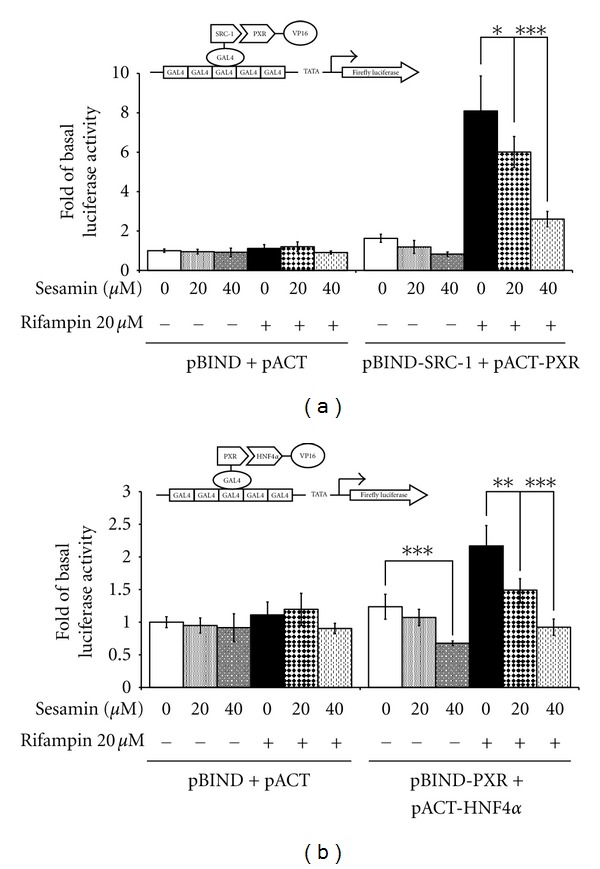
Disruption of protein-protein interactions between PXR and SRC-1/HNF4*α* in the presence of sesamin in a mammalian two-hybrid assay. Mammalian two-hybrid studies were conducted in LS174T cells to elucidate the effects of sesamin on the interactions between (a) SRC-1 receptor-interacting domain (RID) (pBIND-SRC-1); (b) full-length HNF4*α* (pACT-HNF4*α*), and the full-length clone of PXR (pACT-PXR and pBIND-PXR, resp.). The schematic diagram of the plasmids used is illustrated at the left top of each figure. The cells were harvested in equal aliquots at 24 h to determine luciferase activity. All transfections were normalized for transfection efficiency by *β*-galactosidase activity. The results are expressed as mean ± SD (*n* = 4); *P < 0.05; **P < 0.01; and ***P < 0.001, as compared to DMSO-treated or 20 *μ*M rifampin-treated cells, as indicated.

As shown in Figure 8(a), in the absence of rifampin, 20 and 40 *μ*M sesamin decreased the interaction between PXR and SRC-1 by 27% and 49.1% (*P* < 0.05 and *P* < 0.001), as compared to control groups in LS174T cells. In contrast, rifampin strongly promoted the specific interaction between PXR and SRC-1 by 8.1 folds; sesamin attenuated the interaction between PXR and SRC-1 concentration-dependent manner, with a 25.8% (*P* < 0.05) and 67.9% (*P* < 0.001) inhibition at 20 and 40 *μ*M sesamin, respectively.

The interaction between HNF4*α* and PXR was also disrupted by sesamin. As shown in Figure 8(b), the interaction of cells transfected with pBIND-PXR and pACT-HNF4*α*, without rifampin induction, decreased by 45.2% (*P* < 0.001) compared to DMSO-treated cells. Rifampin enhanced the protein-protein interaction of PXR and HNF4*α* by 2.2 folds. As expected, sesamin disrupted this interaction in a concentration-dependent manner. A decrease of 31.3% was seen in cells exposed to 20 *μ*M sesamin (*P* < 0.01), and a decrease of 57.6% was seen in cells exposed to 40 *μ*M sesamin (*P* < 0.001), as compared to rifampin-treated groups which were not exposed to sesamin. These data suggest that sesamin inhibits SRC-1/PXR and HNF4*α*/PXR interactions, thus transactivation in the regulation of CYP3A4 gene expression occurs through the transcriptional control.

### 3.8. CYP3A4 Promoter Activity in the Presence of Sesamin and Paclitaxel or SR12813

**Figure 9:**
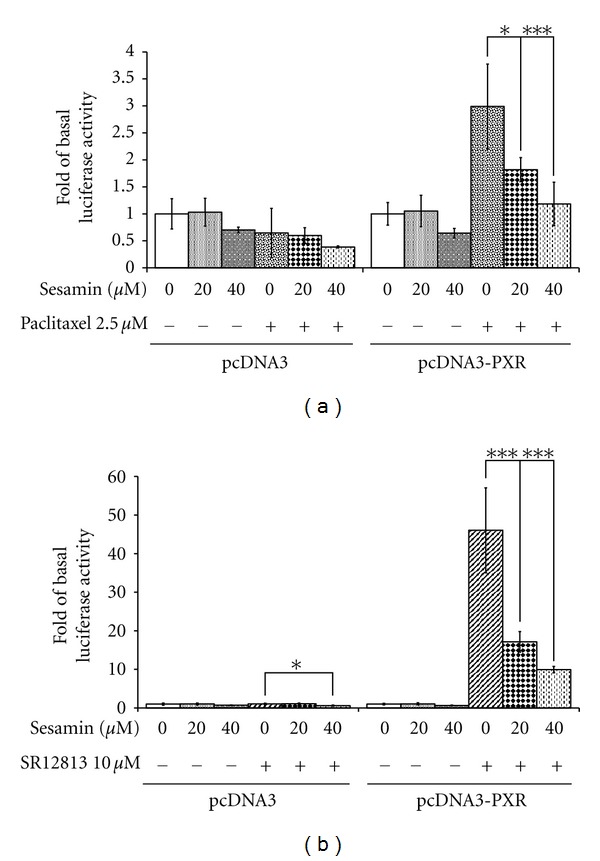
Downregulation of CYP3A4 reporter activity on PXR agonist, paclitaxel- and SR12813-mediated induction by sesamin. LS174T cells were cotransfected with a vector control (pcDNA3) or PXR expression plasmid (pcDNA3-PXR), a CYP3A4 luciferase reporter plasmid, and a pRC-CMV-*β*-galactosidase vector. Subsequently, the transfected cells were exposed to sesamin and/or (a) 2.5 *μ*M paclitaxel or (b) 10 *μ*M SR12813 for 24 h. Luciferase activity was measured and normalized to the corresponding *β*-galactosidase activity. The results are expressed as mean ± SD (*n* = 4); *P < 0.05 and ***P < 0.001, as compared to rifampin-treated cells, as indicated.

 Because many antineoplastic drugs have a narrow therapeutic index, the ability of drugs (e.g., paclitaxel) to activate PXR may be limited because of autoinduced metabolism [56]. This implies that sesamin antagonism of PXR activity might improve the pharmacokinetic properties of these drugs. As shown in Figure 9(a), 2.5 *μ*M paclitaxel enhanced CYP3A4 promoter activity in LS174T cells up to 3 folds as compared to the controls. When the cells were cotreated with paclitaxel and 20 or 40 *μ*M sesamin, CYP3A4 promoter activity reduced by 39.1% (*P* < 0.05) and 60.5% (*P* < 0.001), respectively, when compared with paclitaxel-induced controls. These results indicated that sesamin may rescue PXR agonists from autoinduced metabolism.

SR12813, the most efficient PXR agonist discovered to date [57], was used in some evaluations to exclude the possibility of rifampin-specific effects. In the presence of 10 *μ*M SR12813, CYP3A4 promoter activity in LS174T cells was enhanced by 46.1 folds, as compared to DMSO-treated cells (Figure 9(b)). The presence of sesamin (20 or 40 *μ*M) resulted in 62.8% (*P* < 0.001) or 78.4% (*P* < 0.001) inhibition, respectively, of transcriptional activity induced by SR12813. The results showed that inhibition of CYP3A4 by sesamin was not a rifampin-specific effect.

### 3.9. Sesamin Increases Gene Expression of Enzymes Involved in Phase II Metabolism

**Figure 10:**
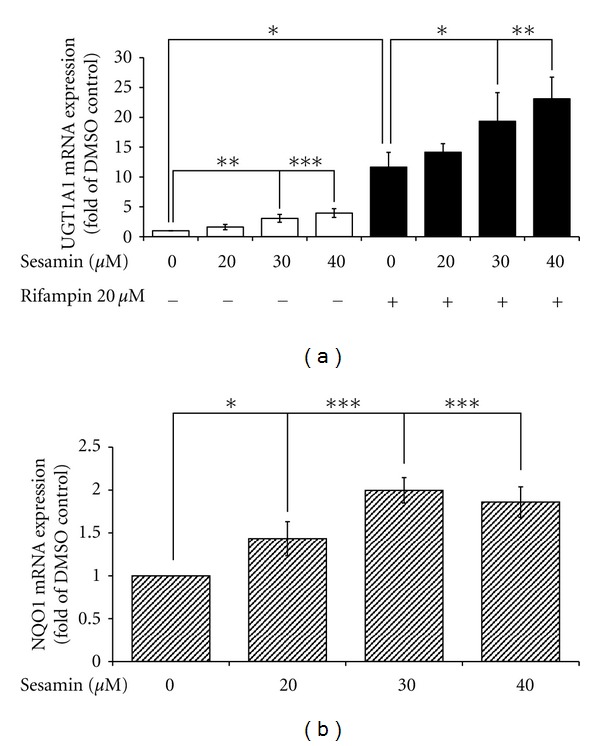
Sesamin-related changes in gene expression of the phase II enzyme gene (*UGT1A1*) and the Nrf2 target gene (*NQO1*). HepG2 cells were treated with sesamin and rifampin, independently or in combination for 48 h; mRNA was collected, and the expression of (a) UGT1A1 and (b) NQO1 were analyzed by RT-PCR and normalized to the corresponding *β*-actin expression. The expression in DMSO-treated controls was set at 1. The results are expressed as mean ± SD (*n* = 3). *P < 0.05; **P < 0.01; and ***P < 0.001, as compared to DMSO-treated or 20-*μ*M rifampin-treated cells, as indicated in the figure.

The discovery that sesamin inhibits CYP3A4 expression led to an investigation of sesamin's effect on the expression of UGT1A1 mRNA in HepG2 cells by RT-PCR. UGT1A1 is an enzyme involved in hepatic phase II metabolism that is also regulated by PXR [58]. As shown in Figure 10(a), sesamin (20 to 40 *μ*M) increased UGT1A1 mRNA expression in HepG2 cells in a concentration-dependent manner, resulting in a 3.1-fold (*P* < 0.01) or 4-fold (*P* < 0.001) increase at sesamin concentrations of 30 and 40 *μ*M, respectively. The expression of UGT1A1 mRNA also increased 11.7 folds in the presence of 20 *μ*M rifampin, consistent with the results of previous studies [59]. With the addition of 20 to 40 *μ*M sesamin, in combination with 20 *μ*M rifampin, expression of UGT1A1 mRNA increased with increasing concentrations of sesamin. This increase was particularly notable at sesamin concentrations of 30 *μ*M, where mRNA levels increased by 65.6% (*P* < 0.05), and 40 *μ*M, where mRNA levels increased by 98% (*P* < 0.01). Presumably, sesamin induced UGT1A1 gene expression through a PXR-independent pathway and may be a nuclear factor E2-related factor 2- (Nrf2-) dependent pathway [60]. Sesamin induced the expression of another Nrf2 target gene, NAD(P)H quinone oxidoreductase 1 (NQO1), in HepG2 cells (Figure 10(b)). In this case, mRNA expression was increased by 1.4 folds (*P* < 0.05) in the presence of 20 *μ*M sesamin, and 2-fold (*P* < 0.001) at a 30 *μ*M sesamin concentration. The net inductive effect of sesamin on UGT1A1 gene expression is interesting because UGT1A1 is also regulated, in part, by PXR.

## 4. Discussion

In this study, sesamin was demonstrated to antagonize the induction of CYP3A4 in HepG2 and LS174T cells. It was also determined to be an inhibitor of rat and human PXR in nuclear receptor transactivation assays. Additionally, sesamin was demonstrated to suppress human CAR activation. Furthermore, the underlying mechanisms of human PXR inhibition were explored. Sesamin was found to inhibit coregulation between SRC-1 and HNF4*α* by disrupting their recruitment and interaction with PXR. These changes were not due to reduced protein levels of those nuclear receptors or coregulators. The inductive effects of SR12813 and paclitaxel could be attenuated by coadministration with sesamin. These results suggest a potential mechanism for preventing inducer-drug interactions between PXR ligands and minimizing the potential for P450 inducer-drug interactions. Although numerous human PXR activators have been identified, to date there are few reports of potent inhibitors of PXR. Sesamin is a novel functional inhibitor of PXR and may be a useful chemical tool for modulating PXR-regulated gene expression *in vitro* or *in vivo*.

Inappropriate PXR activation leads to important and undesirable pathophysiologic consequences [56]. One of PXR's main target genes is *CYP3A4*, a cytochrome that is variably expressed in the liver and small intestine cells [19]. Drug-induced activation of CYP3A4 may affect the safety and effective dosing of narrow therapeutic index chemotherapeutic agents, if they are CYP3A4 substrates. The genetics of CYP3A4 have not explained the variable expression of this cytochrome. This has raised the possibility that individual differences in exposure to dietary or endogenous agents may modulate CYP3A4 transcription, contributing to functional CYP3A4 variability [33]. In this study, sesamin was found to decrease constitutive CYP3A4 activity, as well as mRNA and protein expression. Moreover, it attenuated rifampin-, paclitaxel-, and SR12813-mediated CYP3A4 induction in human hepatoma and colon adenocarcinoma cells. These results suggest that dietary exposure to sesamin could potentially contribute to the large variability in basal CYP3A4 expression between individuals.

 Extensive cross-talk occurs between members of the nuclear receptor superfamily. Both PXR and CAR are closely related nuclear receptors belonging to the nuclear receptor subfamily (NR1) that are involved in CYP3A4 regulation and are endogenously expressed in HepG2 cells [55]. Their ligand-binding domains (LBDs), which share 40% amino acid sequence similarity, bind overlapping areas of the CYP3A4 promoter, but with differential preference [34]. Results from cell-based nuclear receptor transactivation assays indicated that sesamin not only strongly antagonizes PXR but also greatly attenuates CAR-mediated CYP3A4 promoter activity induced by CITCO. Moreover, previous reports have shown that PXR and CAR exhibit promiscuous, low-affinity ligand binding characteristics [53]. Hence, PXR and CAR appear to share similar mechanisms of CYP3A4 regulation.

The full activity of NRs depends on a large number of cellular factors that called coactivator, that do not bind DNA directly, but are selectively recruited to the promoter by the NRs through protein-protein interactions [61]. This suggests the potential activation of NR-regulated genes at the transcriptional levels. A probable mechanism by which sesamin attenuates rifampin-induced CYP3A4 expression is through inhibition of the interaction between PXR and SRC-1. In the present study, sesamin was found to significantly attenuate rifampin-induced interactions between PXR and SRC-1 in a concentration-dependent manner. This suggests that sesamin may inhibit PXR coactivator recruitment, thereby potentially preventing ligand-mediated PXR transcriptional activation of target genes.

HNF4*α* also plays an essential role in the PXR-dependent induction of human CYP3A4 [55, 62]. Thus, an HNF4*α* element adjacent to the PXR- and CAR-binding sites in the human CYP3A4 promoter is an obligatory component of a xenobiotic-responsive distal enhancer [55]. Previous studies revealed that ketoconazole inhibition of CYP3A4 expression not only involves the recruitment of SRC-1, but also HNF4*α* [50, 63]. Analysis of the molecular mechanism of gene transcription inhibition demonstrated that sesamin specifically disrupts the interaction of PXR with HNF4*α* and the coactivator, SRC-1.

Differential activation of PXR orthologs between species has been well reported [64]. Moreover, PXR and CYP3A display cell-type and species-specific differences. Due to the structural differences in its ligand-binding pockets, ligand-mediated activation of PXR can occur through a wide variety of compounds in a species-dependent manner [64]. Sesamin was tested in transient transfection assays using rat PXR and was found to strongly attenuate rat PXR-mediated CYP3A4 promoter activity induced by PCN. The results indicate that sesamin does not have a species-specific effect on human and rat PXR. The inductive effects of paclitaxel and SR12813 on CYP3A4 were also attenuated by coadministration with sesamin, indicating it was not a rifampin-specific effect. The protein expression of PXR and the coregulators RXR*α* and HNF4*α* were not affected by sesamin.

Literature reports indicate that UGT1A1 is transcriptionally regulated by both PXR and CAR through DR-3 and NR-1 elements in the 5′-promoter region [58, 65]. Results from the present study indicated that although sesamin antagonized PXR activity, its induction of UGT1A1 expression appeared to be through a PXR-independent pathway. The expression of UGT1A1 enzymes is regulated by *Nrf2*, which binds to AREs present within the promoter [66]. The result of this binding is an upregulation of the transcription of those genes. We examined the expression of another *Nrf2* target gene, NQO1, as well [66]. Sesamin also significantly induced the mRNA expression of this gene, adding further evidence that sesamin induced UGT1A1 expression primarily through an *Nrf2*-dependent pathway.

Aside from its important role in xenobiotic defense, evidence suggests that during carcinogenesis, PXR promotes tumor cell growth [67]. Cancer chemotherapy normally involves the coadministrated of multiple drugs, and these may have somewhat unpredictable therapeutic outcomes as a result of individual differences in PXR-mediated transcriptional effects. Therefore, a PXR inhibitor can be used to develop a unique method for controlling drug-induced metabolism during chemotherapy, especially if the inhibitor is comparatively nontoxic. Studies have shown that the antineoplastic agent trabectedin ET-743 [45], the antifungal agent ketoconazole(s) [50, 63], the dietary isothiocyanate sulforaphane (SFN) [44], the human HIV protease inhibitor, A-792611 [68], the phytoestrogen coumestrol [46], a topoisomerase I inhibitors camptothecin (CPT) [47], and metformin [69] are all functional inhibitors of PXR.

A genotoxicity report from Hori et al. [70] included analysis of the plasma levels of sesamin in Crlj:CD1 (ICR) mice 3 h after the second of 2 doses, administered orally 24 h apart, of sesamin at 0.5, 1.0, and 2.0 g/kg of body weight. The plasma levels of sesamin were 2.11, 8.18, and 8.00 *μ*g/mL, or approximately 6, 23.1, and 22.6 *μ*M, respectively. Our results indicate that 20 *μ*M sesamin is sufficient to decrease CYP3A4 activity and mRNA and protein expression levels significantly. Therefore, a plasma concentration of 20 *μ*M sesamin, which is sufficient to modulate CYP3A4 according to our *in vitro* data, may well be achievable by oral ingestion, at least in animal studies. Differences in genetics, geographical origin, growing conditions, seed size, and capsule position may contribute to the variable amounts of sesamin in seeds. Rangkadilok et al. [71] showed ranges of sesamin content of 0.05–4.71 mg/g of seed in the landrace black seed lines and 0.74–7.23 mg/g of seed in white sesame seeds. The sesamin content of Taiwanese sesame oil is 9.47 ± 2.28 mg/g of oil [72]. Here, we describe a novel, effective PXR inhibitor from a naturally occurring substance, sesamin, and we believe that another PXR inhibitor with higher efficiency but lower toxicity can be found on the same lines.

HepaRG cells, which were derived from a female hepatocarcinoma patient, are capable of differentiating into biliary epithelial cells and hepatocytes [73]. They retain many DMEs activities and upregulate mRNA expression of such enzymes in the presence of xenobiotics. This ability is in stark contrast to that of the frequently used HepG2 cells, and HepaRG cells have, therefore, been touted as better surrogates for primary human hepatocytes for drug metabolism and disposition studies. In this study, we continued to use HepG2 cells as a simple model system that avoids the interindividual and interspecies variations and subsequent interpretation difficulties that complicate *in vivo *approaches. Moreover, these cells are known to constitutively express CYP1A1/2, 2A6, 2B6, 2C9/19, 2D6, 2E1, and 3A4/5 [74]. Researchers using a cell-based PXR reporter assay to measure possible CYP3A4 induction/inhibition in HepG2 cells showed *CYP3A4 *mRNA expression and enzymatic activity levels similar to those in cultured primary human hepatocytes [74]. Therefore, this cell line remains an excellent surrogate for primary human hepatocytes for studying induction of CYP450s. Our results demonstrate that PXR inducers still significantly enhance the activity and mRNA and protein expression levels of CYP3A4 in HepG2 cells, implying that, for several compounds, the CYP activities in HepG2 cells are high enough to activate xenobiotic receptors and thereby induce further CYP expression.

In conclusion, we have identified a naturally occurring lignan, sesamin, that is a potent inhibitor of human and rat PXR. The study provides evidence that sesamin attenuates the induction of CYP3A4 by inhibiting agonist-activated PXR and CAR. Sesamin disrupts the interaction of PXR with SRC-1 and HNF4*α* without affecting expression of NR-related modulators. These findings suggest a complementary mechanism by which ingestion of this naturally occurring phytochemical may reduce the frequency of adverse drug reactions secondary to PXR-mediated induction of drug clearance via CYP3A4. This may improve the therapeutic efficacy of certain drugs and may also play a role in reducing the formation of CYP3A4-mediated reactive metabolites.

## Figures and Tables

**Table 1:** Primer sequences used for real-time polymerase chain reaction.

Gene		Primer sequence (5′–3′)	PCR condition
CYP3A4	(F)	5′-GGG AAG CAG AGA CAG GCA AG-3′	Denaturation (30 s at 95°C)
	(R)	5′-GAG CGT TTC ATT CAC CAC CA-3′	
UGT1A1	(F)	5′-TTT TGT CTG GCT GTT CCC ACT-3′	Annealing (30 s at 60°C)
	(R)	5′-GAA GGT CAT GT GAT CTG AAT GAG A-3′	
NQO1	(F)	5′-CCA TTC TGA AAG GCT GGT TTG-3′	Extension (30 s at 72°C)
	(R)	5′-CTA GCT TTG ATC TGG TTG TC-3′	
*β*-actin	(F)	5′-GTG GGG CGC CCC AGG CAC CA-3′	45 cycles
	(R)	5′-CAC CCC GCG GGG TCC GTG GT-3′	

(F): forward primer; (R): reverse primer.

## References

[1] Hirose N, Inoue T, Nishihara K (1991). Inhibition of cholesterol absorption and synthesis in rats by sesamin. *Journal of Lipid Research*.

[2] Ogawa H, Sasagawa S, Murakami T, Yoshizumi H (1995). Sesame lignans, modulate cholesterol metabolism in the stroke-prone spontaneously hypertensive rat. *Clinical and Experimental Pharmacology and Physiology*.

[3] Hirata F, Fujita K, Ishikura Y, Hosoda K, Ishikawa T, Nakamura H (1996). Hypocholesterolemic effect of sesame lignan in humans. *Atherosclerosis*.

[4] Kiso Y (2004). Antioxidative roles of sesamin, a functional lignan in sesame seed, and it’s effect on lipid- and alcohol-metabolism in the liver: A DNA microarray study. *BioFactors*.

[5] Rogi T, Tomimori N, Ono Y, Kiso Y (2011). The mechanism underlying the synergetic hypocholesterolemic effect of sesamin and *α*-tocopherol in rats fed a high-cholesterol diet. *Journal of Pharmacological Sciences*.

[6] Fukuda N, Miyagi C, Zhang L (1998). Reciprocal effects of dietary sesamin on ketogenesis and triacylglycerol secretion by the rat liver. *Journal of Nutritional Science and Vitaminology*.

[7] Matsumura Y, Kita S, Morimoto S (1995). Antihypertensive effect of sesamin. I. Protection against deoxycorticosterone acetate-salt-induced hypertension and cardiovascular hypertrophy. *Biological and Pharmaceutical Bulletin*.

[8] Miyawaki T, Aono H, Toyoda-Ono Y, Maeda H, Kiso Y, Moriyama K (2009). Antihypertensive effects of sesamin in humans. *Journal of Nutritional Science and Vitaminology*.

[9] Noguchi T, Ikeda K, Sasaki Y (2001). Effects of vitamin E and sesamin on hypertension and cerebral thrombogenesis in stroke-prone spontaneously hypertensive rats. *Hypertension Research*.

[10] Yamashita K, Nohara Y, Katayama K, Namiki M (1992). Sesame seed lignans and *γ*-tocopherol act synergistically to produce vitamin E activity in rats. *Journal of Nutrition*.

[11] Chavali SR, Zhong WW, Utsunomiya T, Forse RA (1997). Decreased production of interleukin-1-beta, prostaglandin-E2 and thromboxane-B2, and elevated levels of interleukin-6 and -10 are associated with increased survival during endotoxic shock in mice consuming diets enriched with sesame seed oil supplemented with Quil-A saponin. *International Archives of Allergy and Immunology*.

[12] Chavali SR, Forse RA (1999). Decreased production of interleukin-6 and prostaglandin E2 associated with inhibition of Δ-5 desaturation of *ω*6, fatty acids in mice fed safflower oil diets supplemented with sesamol. *Prostaglandins Leukotrienes and Essential Fatty Acids*.

[13] Hirose N, Doi F, Ueki T (1992). Suppressive effect of sesamin against 7,12-Dimethylbenz[a]anthracene induced rat mammary carcinogenesis. *Anticancer Research*.

[14] Hibasami H, Fujikawa T, Takeda H (2000). Induction of apoptosis by Acanthopanax senticosus HARMS and its component, sesamin in human stomach cancer KATO III cells. *Oncology Reports*.

[15] Miyahara Y, Komiya T, Katsuzaki H (2000). Sesamin and episesamin induce apoptosis in human lymphoid leukemia Molt 4B cells. *International journal of molecular medicine*.

[16] Harikumar KB, Sung B, Tharakan ST (2010). Sesamin manifests chemopreventive effects through the suppression of NF-*κ*B-regulated cell survival, proliferation, invasion, and angiogenic gene products. *Molecular Cancer Research*.

[17] Lee CC, Liu KJ, Wu YC, Lin SJ, Chang CC, Huang TS (2011). Sesamin inhibits macrophage-induced vascular endothelial growth factor and matrix metalloproteinase-9 expression and proangiogenic activity in breast cancer cells. *Inflammation*.

[18] Tanabe H, Kuribayashi K, Tsuji N, Tanaka M, Kobayashi D, Watanabe N (2011). Sesamin induces autophagy in colon cancer cells by reducing tyrosine phosphorylation of EphA1 and EphB2. *International Journal of Oncology*.

[19] Guengerich FP (1999). Cytochrome P-450 3A4: regulation and role in drug metabolism. *Annual Review of Pharmacology and Toxicology*.

[20] Shimada T, Yamazaki H, Mimura M, Inui Y, Guengerich FP (1994). Interindividual variations in human liver cytochrome P-450 enzymes involved in the oxidation of drugs, carcinogens and toxic chemicals: Studies with liver microsomes of 30 Japanese and 30 Caucasians. *Journal of Pharmacology and Experimental Therapeutics*.

[21] Paine MF, Hart HL, Ludington SS, Haining RL, Rettie AE, Zeldin DC (2006). The human intestinal cytochrome P450 ‘pie’. *Drug Metabolism and Disposition*.

[22] Rendic S (2002). Summary of information on human CYP enzymes: human P450 metabolism data. *Drug Metabolism Reviews*.

[23] Kamdem LK, Meineke I, Gödtel-Armbrust U, Brockmöller J, Wojnowski L (2006). Dominant contribution of P450 3A4 to the hepatic carcinogenic activation of aflatoxin B1. *Chemical Research in Toxicology*.

[24] Kale VM, Miranda SR, Wilbanks MS, Meyer SA (2008). Comparative cytotoxicity of alachlor, acetochlor, and metolachlor herbicides in isolated rat and cryopreserved human hepatocytes. *Journal of Biochemical and Molecular Toxicology*.

[25] Moore LB, Goodwin B, Jones SA (2000). St. John’s wort induces hepatic drug metabolism through activation of the pregnane X receptor. *Proceedings of the National Academy of Sciences of the United States of America*.

[26] Özdemir V, Kalow W, Tang BK (2000). Evaluation of the genetic component of variability in CYP3A4 activity: a repeated drug administration method. *Pharmacogenetics*.

[27] Rochat B (2005). Role of cytochrome P450 activity in the fate of anticancer agents and in drug resistance: focus on tamoxifen, paclitaxel and imatinib metabolism. *Clinical Pharmacokinetics*.

[28] Ohno Y, Hisaka A, Ueno M, Suzuki H (2008). General framework for the prediction of oral drug interactions caused by CYP3A4 induction from in vivo information. *Clinical Pharmacokinetics*.

[29] Kliewer SA, Moore JT, Wade L (1998). An orphan nuclear receptor activated by pregnanes defines a novel steroid signaling pathway. *Cell*.

[30] Goodwin B, Hodgson E, Liddle C (1999). The orphan human pregnane X receptor mediates the transcriptional activation of CYP3A4 by rifampicin through a distal enhancer module. *Molecular Pharmacology*.

[31] Lehmann JM, McKee DD, Watson MA, Willson TM, Moore JT, Kliewer SA (1998). The human orphan nuclear receptor PXR is activated by compounds that regulate CYP3A4 gene expression and cause drug interactions. *Journal of Clinical Investigation*.

[32] Bertilsson G, Heidrich J, Svensson K (1998). Identification of a human nuclear receptor defines a new signaling pathway for CYP3A induction. *Proceedings of the National Academy of Sciences of the United States of America*.

[33] Blumberg B, Sabbagh W, Juguilon H (1998). SXR, a novel steroid and xenobiotic-sensing nuclear receptor. *Genes and Development*.

[34] Wei P, Zhang J, Dowhan DH, Han Y, Moore DD (2002). Specific and overlapping functions of the nuclear hormone receptors CAR and PXR in xenobiotic response. *Pharmacogenomics Journal*.

[35] Maglich JM, Stoltz CM, Goodwin B, Hawkins-Brown D, Moore JT, Kliewer SA (2002). Nuclear pregnane X receptor and constitutive androstane receptor regulate overlapping but distinct sets of genes involved in xenobiotic detoxification. *Molecular Pharmacology*.

[36] Pascussi JM, Robert A, Nguyen M (2005). Possible involvement of pregnane X receptor-enhanced CYP24 expression in drug-induced osteomalacia. *Journal of Clinical Investigation*.

[37] Sonoda J, Rosenfeld JM, Xu L, Evans RM, Xie W (2003). A nuclear receptor-mediated xenobiotic response and its implication in drug metabolism and host protection. *Current Drug Metabolism*.

[38] Kast HR, Goodwin B, Tarr PT (2002). Regulation of multidrug resistance-associated protein 2 (ABCC2) by the nuclear receptors pregnane X receptor, farnesoid X-activated receptor, and constitutive androstane receptor. *Journal of Biological Chemistry*.

[39] Staudinger JL, Madan A, Carol KM, Parkinson A (2003). Regulation of drug transporter gene expression by nuclear receptors. *Drug Metabolism and Disposition*.

[40] Kliewer SA, Goodwin B, Willson TM (2002). The nuclear pregnane X receptor: a key regulator of xenobiotic metabolism. *Endocrine Reviews*.

[41] Kawana K, Ikuta T, Kobayashi Y, Gotoh O, Takeda K, Kawajiri K (2003). Molecular mechanism of nuclear translocation of an orphan nuclear receptor, SXR. *Molecular Pharmacology*.

[42] Chen Y, Tang Y, Wang MT, Zeng S, Nie D (2007). Human pregnane X receptor and resistance to chemotherapy in prostate cancer. *Cancer Research*.

[43] Wang H, Huang H, Li H (2007). Activated pregnenolone X-receptor is a target for ketoconazole and its analogs. *Clinical Cancer Research*.

[44] Zhou C, Poulton EJ, Grün F (2007). The dietary isothiocyanate sulforaphane is an antagonist of the human steroid and xenobiotic nuclear receptor. *Molecular Pharmacology*.

[45] Synold TW, Dussault I, Forman BM (2001). The orphan nuclear receptor SXR coordinately regulates drug metabolism and efflux. *Nature Medicine*.

[46] Wang H, Li H, Moore LB (2008). The phytoestrogen coumestrol is a naturally occurring antagonist of the human pregnane X receptor. *Molecular Endocrinology*.

[47] Chen Y, Tang Y, Robbins GT, Nie D (2010). Camptothecin attenuates cytochrome P450 3A4 induction by blocking the activation of human pregnane X receptor. *Journal of Pharmacology and Experimental Therapeutics*.

[48] Lim YP, Liu CH, Shyu LJ, Huang JD (2005). Functional characterization of a novel polymorphism of pregnane X receptor, Q158K, in Chinese subjects. *Pharmacogenetics and Genomics*.

[49] Lim YP, Huang JD (2007). Pregnane X receptor polymorphism affects CYP3A4 induction via a ligand-dependent interaction with steroid receptor coactivator-1. *Pharmacogenetics and Genomics*.

[50] Lim YP, Kuo SC, Lai ML, Huang JD (2009). Inhibition of CYP3A4 expression by ketoconazole is mediated by the disruption of pregnane X receptor, steroid receptor coactivator-1, and hepatocyte nuclear factor 4*α* interaction. *Pharmacogenetics and Genomics*.

[51] Rigalli JP, Ruiz ML, Perdomo VG, Villanueva SSM, Mottino AD, Catania VA (2011). Pregnane X receptor mediates the induction of P-glycoprotein by spironolactone in HepG2 cells. *Toxicology*.

[52] Wang H, Venkatesh M, Li H (2011). Pregnane X receptor activation induces FGF19-dependent tumor aggressiveness in humans and mice. *Journal of Clinical Investigation*.

[53] Kliewer SA, Willson TM (2002). Regulation of xenobiotic and bile acid metabolism by the nuclear pregnane X receptor. *Journal of Lipid Research*.

[54] Li T, Chiang JYL (2006). Rifampicin induction of CYP3A4 requires pregnane X receptor cross talk with hepatocyte nuclear factor 4*α* and coactivators, and suppression of small heterodimer partner gene expression. *Drug Metabolism and Disposition*.

[55] Tirona RG, Lee W, Leake BF (2003). The orphan nuclear receptor HNF4*α* determines PXR- and CAR-mediated xenobiotic induction of CYP3A4. *Nature Medicine*.

[56] Harmsen S, Meijerman I, Beijnen JH, Schellens JHM (2007). The role of nuclear receptors in pharmacokinetic drug-drug interactions in oncology. *Cancer Treatment Reviews*.

[57] Watkins RE, Wisely GB, Moore LB (2001). The human nuclear xenobiotic receptor PXR: structural determinants of directed promiscuity. *Science*.

[58] Sugatani J, Sueyoshi T, Negishi M, Miwa M (2005). Regulation of the human UGT1A1 gene by nuclear receptors constitutive active/androstane receptor, pregnane X receptor, and glucocorticoid receptor. *Methods in Enzymology*.

[59] Zhou C, Tabb MM, Sadatrafiei A, Grün F, Blumberg B (2004). Tocotrienols activate the steroid and xenobiotic receptor, SXR, and selectively regulate expression of its target genes. *Drug Metabolism and Disposition*.

[60] Zhang DD (2006). Mechanistic studies of the Nrf2-Keap1 signaling pathway. *Drug Metabolism Reviews*.

[61] Glass CK, Rosenfeld MG (2000). The coregulator exchange in transcriptional functions of nuclear receptors. *Genes and Development*.

[62] Lim YP, Huang JD (2008). Interplay of pregnane X receptor with other nuclear receptors on gene regulation. *Drug Metabolism and Pharmacokinetics*.

[63] Huang H, Wang H, Sinz M (2007). Inhibition of drug metabolism by blocking the activation of nuclear receptors by ketoconazole. *Oncogene*.

[64] Moore LB, Maglich JM, McKee DD (2002). Pregnane X receptor (PXR), constitutive androstane receptor (CAR), and benzoate X receptor (BXR) define three pharmacologically distinct classes of nuclear receptors. *Molecular Endocrinology*.

[65] Xie W, Yeuh MF, Radominska-Pandya A (2003). Control of steroid, heme, and carcinogen metabolism by nuclear pregnane X receptor and constitutive androstane receptor. *Proceedings of the National Academy of Sciences of the United States of America*.

[66] Yueh MF, Tukey RH (2007). Nrf2-Keap1 Signaling pathway regulates human UGT1A1 expression in vitro and in transgenic UGT1 mice. *Journal of Biological Chemistry*.

[67] Gupta D, Venkatesh M, Wang H (2008). Expanding the roles for pregnane X receptor in cancer: proliferation and drug resistance in ovarian cancer. *Clinical Cancer Research*.

[68] Healan-Greenberg C, Waring JF, Kempf DJ, Blomme EAG, Tirona RG, Kim RB (2008). A human immunodeficiency virus protease inhibitor is a novel functional inhibitor of human pregnane X receptor. *Drug Metabolism and Disposition*.

[69] Krausova L, Stejskalova L, Wang H (2011). Metformin suppresses pregnane X receptor (PXR)-regulated transactivation of CYP3A4 gene. *Biochemical Pharmacology*.

[70] Hori H, Takayanagi T, Kamada Y (2011). Genotoxicity evaluation of sesamin and episesamin. *Mutation Research*.

[71] Rangkadilok N, Pholphana N, Mahidol C (2010). Variation of sesamin, sesamolin and tocopherols in sesame (Sesamum indicum L.) seeds and oil products in Thailand. *Food Chemistry*.

[72] Wu WH (2007). The contents of lignans in commercial sesame oils of Taiwan and their changes during heating. *Food Chemistry*.

[73] Guillouzo A, Corlu A, Aninat C, Glaise D, Morel F, Guguen-Guillouzo C (2007). The human hepatoma HepaRG cells: a highly differentiated model for studies of liver metabolism and toxicity of xenobiotics. *Chemico-Biological Interactions*.

[74] Westerink WMA, Schoonen WGEJ (2007). Cytochrome P450 enzyme levels in HepG2 cells and cryopreserved primary human hepatocytes and their induction in HepG2 cells. *Toxicology in Vitro*.

